# Case Studies of Severe Microfilaremia in Four Dogs Naturally Infected With *Dirofilaria repens* as the Primary Disease or a Disease Complicating Factor

**DOI:** 10.3389/fvets.2020.577466

**Published:** 2020-09-22

**Authors:** Magdalena E. Wysmołek, Maciej Klockiewicz, Małgorzata Sobczak-Filipiak, Ewa Długosz, Marcin Wiśniewski

**Affiliations:** ^1^Division of Parasitology and Parasitic Diseases, Department of Pre-Clinical Sciences, Institute of Veterinary Medicine, Warsaw University of Life Sciences, Warsaw, Poland; ^2^Department of Pathology and Veterinary Diagnostics, Institute of Veterinary Medicine, Warsaw University of Life Sciences, Warsaw, Poland

**Keywords:** dog, dirofilariosis, microfilaremia, immunology, natural Dirofilaria repens infection

## Abstract

Subcutaneous dirofilariosis in dogs, caused by *Dirofilaria repens*, is an underdiagnosed disease, now recognized for its zoonotic potential, and growing distribution and prevalence across Europe and Asia. Our understanding of the pathogenicity in human and canine host remains unclear, but case reports suggest that microfilariae (Mf) as well as adult *D. repens* may directly cause internal organs damage or may be a factor complicating the course of other ailments. The purpose of the study was to report high Mf in dogs and to discuss potential relevance with co-morbidity. Our data from a modified Knott's test performed on 62 infected dogs indicate that the median Mf count in *D. repens* infections is 675 Mf/ml and we consider microfilaremia above 10,000 Mf/ml as high intensity. This collection of case reports discusses 4 cases of high intensity *D. repens* microfilaremia in companion dogs; one presenting pathology from a very high intensity of adult *D. repens* with post-treatment complications, and 3 dogs in which high microfilaremia was detected incidentally during the management of other primary illnesses. To our knowledge this report describes the highest *D. repens* microfilaremia ever detected in a dog, at 178,000 Mf/ml. The issue of high microfilaremic infections in dogs is poorly studied and there is growing need to identify the presentation and understand the mechanisms of associated pathogenesis in the host-parasite relationship.

## Introduction

*Dirofilaria repens* is a zoonotic filarial nematode transmitted to dogs by a mosquito vector and is the principal agent of human dirofilariosis in the Old World (1). Humans are considered a dead-end, accidental host for *D. repens*, but a growing body of case reports suggests that humans may instead be a dual facultative host in which the parasite can achieve maturity and release microfilariae (Mf) into the bloodstream (2, 3). Recently, there has been documentation in Poland of a human patient having microfilaremia (360 Mf/ml) (4). Furthermore, an ocular mucosa associated lymphoid tissue lymphoma (MALT) has been reported in a human patient in Poland as a possible consequence of dirofilariosis (5). The number of human subcutaneous dirofilariosis cases in Europe has increased in recent years and current estimations in some areas is roughly 10 infected out of 100,000 inhabitants (6). True infection prevalence is likely underestimated because of misdiagnosis and under sampling. Additionally, with increasing globalization and climate change (1, 7), these numbers are expected to continue to grow and, therefore, proper diagnosis and treatment in dogs and humans will be increasingly important for managing the spread and disease burden of *D. repens* infections.

Currently, the treatment recommended by the European Society for Dirofilariosis and Angiostrongylosis (ESDA) for *D. repens* infections consists of 2.5 mg/kg moxidectin (in a spot-on formulation containing also 10 mg/kg imidacloprid) given monthly^1^. However, because so few cases of high intensity *D. repens* microfilaremia have been reported (8), there are no guidelines for its management (1), or rationale for its cause.

The gold standard for diagnosis of subcutaneous dirofilariosis is the modified Knott's test which allows visual detection and morphological identification of Mf (9), and was used to evaluate the Mf burden in all cases described in this article. Our data from a modified Knott's tests performed on 62 infected dogs indicate that the median Mf count in *D. repens* infections is 675 Mf/ml (unpublished data), and the authors consider microfilaremia above 10,000 Mf/ml as being high. Dogs presented in this report all tested positive for having high microfilaremia with the highest level ever reported in *D. repens* infection at 178,000 Mf/ml. Helminths are known to suppress the host immune response in order to establish infection. It is therefore possible that patients presenting with high Mf counts may represent a population with pre-existing immunosuppression and are thus, unable to naturally control or respond to infection (9). All dogs in this study were diagnosed with terminal conditions which might be correlated with an underlying immunodeficiency.

## CASE #1

### Clinical Presentation

A 9-year-old entire male Shepherd dog was admitted to a veterinary clinic on 27/05/2016 due to severe weight loss and two infected purulent wounds in the scrotum area, associated with intensive licking. Physical examination revealed the presence of a chronic bilateral *otitis externa* and an inflammation of the interdigital spaces. Swabs from both ears and interdigital spaces were taken.

### Diagnosis

During a blood examination on 03/09/2016 we detected severe leucocytosis [33,64 G/l (reference value 6–12 G/l)] related to the increase in band neutrophils [10% (reference value 0–6%)] with a mild increase in aspartate aminotransferase [91 U/l (reference value 0–45 U/l)], moderate increase in phosphatase kinase [1,160 U/l (reference value 25–467 U/l)], decrease in albumin [1,8 g/dl (reference value 3,3–5,6 g/dl) and the presence of Mf was observed in the blood smear. Identification of *D. repens* was obtained using multiplex PCR performed using primers designed by Genchi et al. (10). The intensity of microfilaremia was evaluated using a modified Knott's test and revealed 14,512 Mf/ml of blood. The ear swab sample tested positive for *Malassezia pachydermatis*, and the swab from interdigital space was positive for *Pseudomonas aeruginosa*.

### Treatment

During surgical castration on 7/09/2016, 26 adult *D. repens* worms were found in the spermatic cord, testicles and scrotum tissue. The external canals were treated with nystatin according to the antifungal susceptibility test, and a general antibiotic was administered for the dermatitis of interdigital space according to the antibiogram.

After castration and wound excision, the dog started to gain weight. *Otitis externa* as well as the dermatitis were ameliorating after appropriate treatment. Beginning on 24/10/2016, 6 monthly treatments of topical moxidectin with imidacloprid were administered as efficacious treatment against adult *D. repens* in dogs (11). One week after the first application no Mf were detected in the peripheral blood. The body condition of the dog changed from very thin to normal within 4 months. Despite amelioration of the general condition, a necrosis and pyogranuloma of interdigital spaces associated with severe dermatitis of the abdomen and limbs developed with episodes of amelioration and deteriorations between 10/11/2016 and 28/08/2018. A course of antibiotics was administered according to the antibiogram but no improvement was observed. A generalized demodicosis, caused by *Demodex canis*, was diagnosed and, as the declining clinical state of the dog was progressing and no amelioration could be established, the owner did not wish to pursue further treatment. Finally, the patient became anorexic and expired on 15/09/2018. The cause of death reported after necropsy was a perforated stomach ulcer. Moreover, the histopathological examination of internal organs revealed a chronic cardiac insufficiency and pathological changes indicating a chronic inflammation in the kidneys, liver and spleen.

### Discussion

The presence of 26 adult *D. repens* likely caused severe pain and pruritus. It is noteworthy that there were no noticeable behaviors to indicate the extremely high number of Mf circulating in the blood stream. The only obvious clinical and hematological signs were likely associated with presence of a large number of adult worms in the scrotum area. We suspect that the severe dermatitis which appeared post-treatment might have been associated with *Wolbachia* release after *Dirofilaria* spp. adulticide treatment as reported by previous studies in *D. immitis* infections (12–15). Furthermore, German Shepherds are predisposed to primary immunodeficiency (16) and it has been postulated that high microfilaremia might be associated with a state of immunosuppression (17). The chronic yeast infection (usually secondary to an immune disorder in dogs) as well as the appearance of generalized demodicosis are possible indicators of immunodeficiency in this patient (18, 19).

## CASE #2

### Clinical Presentation

A 7-year-old male entire Yorkshire Terrier was admitted to the veterinary clinic on 06/08/2019 due to sudden anorexia, apathy and diarrhea. During the clinical examination the dog presented a hypotonic-hyporesponsiveness concomitant with exophthalmos, severe dehydration, hypothermia, bradycardia and pale mucous membranes. A treatment based on dexamethasone, caffeine, atropine, ceftriaxone and fluid therapy was initiated and a blood sample taken for analysis.

### Diagnosis

Hematology revealed a severe leukocytosis [58.1 × 10^3^/mm^3^ (reference value 6.0–12.0 × 10^3^/mm^3^)] resulting from neutrophilia [54.2 × 10^3^/mm^3^ (reference value 3.00–10.00 10^3^/mm^3^)], and eosinophilia [23.42 × 10^3^/mm^3^ (reference value 0–0.60 10^3^/mm^3^)] with mild anemia [HCT = 39.5% (reference value 44.0–57.0%)], and a severe increase in alkaline phosphatase [551 U/l (reference value 20–150 U/l)], alanine aminotransferase [131 U/l (reference value 10–118 U/l)] and a decrease in albumin [1.5 g/dL (reference value 2.5–4.4 g/dL)]. The glucose level was 99 mg/dl (reference value 80–120 mg/dl) and the modified Knott's test revealed 11,560 Mf/ml. After testing and excluding Canine Coronavirus (CCV), Canine Parvovirus and *Giardia* spp. infections, and according to anamnesis, a diagnosis of poisoning with taxine alkaloid was decided. On 08/08/2019 the state of the patient continued to deteriorate despite intense care therapy, and a decision of euthanasia was made.

### Discussion

It is hard to evaluate the impact of co-infection with *D. repens* in this case, but one can suppose that it may have had a negative influence on the development of the condition, as taxine alkaloids (from yew) can cause lethal poisoning, in particular due to the compound's toxic effect on the cardiovascular apparatus (20). It is certainly possible that the severe microfilaremia might create a blood flow disturbance of capillary circulation by mechanical obstruction. Additionally, it has been observed in other filarial parasitic diseases that dead Mf release toxic products that affect the capillaries (21, 22). Unfortunately, the prognosis in taxine alkaloid poisoning without immediate stomach flushing is usually fatal and the presence of subcutaneous dirofilariosis was not likely influential in the course of the intoxication (20), but may be of concern for chronic anemia.

## CASE #3

### Clinical Presentation

A 10-year-old entire male mix breed dog was admitted to the clinic due to constipation. During the physical examination a testicular asymmetry, perineal hernia, fecal impaction as well as an anal sac tumor of 2 × 2 cm were detected.

### Diagnosis

The blood test revealed moderate anemia [RBC = 4.98 T/l (reference value 5.5–8.5 T/l), HCT = 32.6% (reference value 37.0–55.0%), HGB = 11,1 g/dl (reference value 12–18 g/dl)], severe leucocytosis [41.04 G/l (reference value 6.0–12.0 G/l)] resulted from: neutrophilia [34.06 G/l (reference value 2.9–13.6 G/l)], monocytosis [2.05 G/l (reference value 0.4–1.6 G/l)], eosinophilia [3.69 G/l (reference value 0–3.1 G/l)], thrombocytopenia [19 G/l (reference value 150–500 G/l)] and 40 150 Mf/ml. In addition, a mild increase in alanine aminotransferase [72 U/l (reference value 0–60.0 U/l)] and a decrease of albumin [3.2 g/dl (reference value 3.3–5.6 g/dl)] were detected, and ultrasonography revealed ascites, which was analyzed. The ascites contained Mf, granulocytes and atypical binucleated cells with many mitotic figures suggesting a neoplastic process. The owner did not wish to pursue any treatment nor euthanize and decided to take the patient home.

### Discussion

This is the second report of finding Mf in the body fluid in a dog, the first of which was by Pazdzior-Czapula et al. (23). The issue and association between neoplastic processes and the presence of *D. repens* Mf or adults has been previously observed in cancer of the perianal gland, mast cells, epithelial cells, hemangiopericytoma, and trichoblastoma ribbon in dogs (20), as well as an ocular lymphoma from which the parasite was previously removed from a human patient (5). It has also been reported that parasitic infections or abnormalities of the immune system are at a high risk for developing cancer (5, 24), but the exact relationship between subcutaneous dirofilariosis and neoplastic process is still unknown.

## CASE #4

### Clinical Presentation

A 10-year-old entire Saint Bernard male was admitted to the clinic due to intolerance of any physical activity and permanent dyspnoea. Four months prior, the dog was pursuing therapy in another clinic and received a treatment of benazepril, spironolactone, theophylline, and nitroglycerin with no diagnosis, but anamnesis of increasing fatigue after physical exercise. During our physical examination, dyspnoea and cyanosis of tongue and mucous membranes (hypoxia) were noted.

### Diagnosis

After sedation of the patient, additional diagnostics were performed. Thoracic radiographs revealed a dilation of the esophagus, computed tomography and electrocardiography revealed no alternations. A moderate leucocytosis [16.7 × 10^3^/mm^3^ (reference value 6.0–10.00 x 10^3^/mm^3^)], mild neutrophilia [13.90 x 10^3^/mm^3^ (3.0–10.00 × 10^3^/mm^3^)], and eosinophilia [1.5 × 10^3^/mm^3^ (reference value 0.0–0.60 × 10^3^/mm^3^)] were observed in the hematology, and the biochemistry parameters were all in normal reference ranges. The echocardiographic measurements were within standard normal age limits. The rapid CANIV-4 commercial test was positive for *D. immitis*, but multiplex PCR revealed only the presence of *D. repens*. A modified Knott's test was performed which detected 178,000 Mf/ml and a double morphological identification of *D. repens*, not *D. immitis* was confirmed. As systolic dysfunction was suspected aspirin was administrated which resulted in mild improvement of clinical signs. A few days later echocardiographic examination was performed and no nematodes were detected in the pulmonary artery. The echocardiographic measurements were within standard normal age limits. Only immobilization brought relief to the dog. A final diagnosis of severe laryngeal paralysis was decided upon, and pharmacological treatment was performed, but with no improvement. Before any surgical interventions could be considered the patient died at home from apparent suffocation. The owners did not permit a necropsy.

### Discussion

Laryngeal paralysis is a respiratory disorder that primarily affects older (> 9 years of age), large-breed and giant-breed dogs, such as the Saint Bernard in this case. In many dogs the etiology remains idiopathic, but one possible cause is suggested to involve immune-mediated neuromuscular disease or other immune disorders like systemic lupus erythematosus (25, 26) In humans, a case of a girl treated for systemic lupus erythematosus was diagnosed with concomitant non-human filarial microfilaremic infection. The authors suggested that an immunity disorder might have led to this filarial infection (27). Leucocytosis and eosinophilia are both commonly associated with helminth infections, and neutrophilia can inhibit a Th1 type response, all of which could be expected to be associated with an increased Mf count. However, these numbers alone are very unlikely to facilitate or explain such a high level of microfilaremia. Although it is not possible to come to any conclusions from this case, the incredibly high level of microfilaremia and its co-morbidity with a presentation of symptoms known to be related to compromised immune system function, is highly suggestive of a causal relationship.

## Final Conclusions

The goal of this article was to report the details of four cases of high *D. repens* microfilaremia. Hematological and biochemical findings in dogs infected with *Dirofilaria repens* included anemia, thrombocytopenia, leucocytosis, neutrophilia, eosinophilia, monocytosis and increased values of alkaline phosphatase, aspartate transaminase, alanine aminotransferase, blood urea nitrogen, creatinine, bilirubin as well as hypoalbuminemia, which is in keeping with results described by other authors (28, 29).

In the cases presented here, it is difficult to determine how long the animals were infected for or to evaluate the contribution of subcutaneous dirofilariosis to the progression of morbidity in the presence of concomitant conditions. Due to the severity of the underlying diseases and symptoms, anthelmintic treatment was only administered to one dog seen in this collection of case studies. Even in the presence of a high burden of microfilaria, treatment with a standard dose of moxidectin was sufficient to reduce Mf count below detection 6 days after application. It should be noted that there is a 24-h microfilarial periodicity, which makes the time of sampling a factor influencing the result of a Knott test (30).

We suspect that the severe dermatitis that appeared post-treatment might have been associated with *Wolbachia* release after *Dirofilaria* spp. worm death as reported in *Dirofilaria immitis* infections (12). Recently, a case report of *D. repens* microfilaremic infection in a human showed successful treatment with only doxycycline targeting the *Wolbachia* endosymbiont (31).

All of the *D. repens* infections reported here were diagnosed incidentally while investigating concomitant conditions, suggesting that high intensity microfilaremia *per se* may go undiagnosed. This implies that there is a greater reservoir for human infection than previously suspected, and perhaps warrants increased monitoring in endemic areas, such as the regular use of a modified Knott's test. It might be discussed if high microfilaremic dogs represent an epidemiological risk of increased transmission, as most mosquitoes die when feeding on hosts with microfilaremia superior to 7,500 Mf/ml (21). However, the daily microfilarial periodicity permits the possibility that some mosquitoes can survive ingesting blood even from high microfilaremic hosts. Therefore, anthelmintic treatment, even in the absence of parasitic symptoms, should be administrated.

It remains unclear why in some patients, *D. repens* infections appear asymptomatic, and in other cases Mf are suspected to be responsible for severe renal or liver damage (28). Veterinarians should consider dirofilariosis as a primary or co-existing disease that may modify or mask the symptoms or progression of a co-morbidity. Furthermore, high intensity microfilaremia in a blood samples should be taken as a potential indictor of the presence of an underlying serious pathology.

Personal correspondence with veterinary practitioners suggest a beneficial effect of adding to the moxidectin standard treatment an anticoagulant, corticosteroids, and doxycycline, depending on the severity of the clinical state of the patient infected with *D. repens;* this is also recommended in high risk thrombosis development with heartworm disease (12). As reported in lymphatic filariasis, doxycycline treatment against *Wolbachia* provides clinical improvement in patients with lymphedema (32). It has also been noted in loiasis that high microfilarial loads (30,000–50,000 Mf/ml) can lead to capillary obstruction from damaged parasites after anthelmintic treatment, and adding corticosteroids to the anti-parasitic treatment should be taken into consideration by ESDA and veterinary practitioners in high intensity microfilaremia (22).

In human *Loa loa* infections occurring with microfilaremia >10,000 Mf/ml of blood, encephalopathy following ivermectin has been well-documented. Treatment with microfilaricidal agents such as ivermectin and diethylcarbamazine may provoke the passage of L. loa Mf into the cerebrospinal fluid and precipitate an encephalopathy (33, 34). Those reactions were not yet described in filariosis of dogs, but a case of a human *D. repens* infection concomitant with meningoencephalitis was successfully treated by initiation of anti-helminth and anti-inflammatory medicine (35). This could be an interesting issue to follow and explain in further studies.

Adult parasite burden cannot be determined precisely in helminth infections (36), especially in *D. repens* where the worms may reside in any number of tissue locations. In Case #1 we found 26 adult worms during castration. Most case reports present findings of just one or few adult worms, however, we cannot exclude the possibility that these are under representations of total adult numbers localized in different tissues. It has been previously documented that a dog infected with over 300 adult *D. repens* showed a microfilaremia of 7,780 Mf/ml (28). This suggests that the quantity of adult worms may be only one of the factor determining Mf in the blood stream, and it is very likely that the immune system of the host plays a large role in regulating microfilariae burden, as is observed in other helminth infections (28, 37). The mechanism regulating the burden of Mf in the bloodstream is not known. It is suspected that certain aspects of host-helminth relationship may be beneficial to the host given the proper balance parasite load and humoral response in the host (37). This relationship is beginning to be investigated pertaining to autoimmune disease, and could be of future interest in the study of *D. repens* infections.

Density-dependent processes play a key role in the transmission dynamics of vector-borne diseases (36). The relationship between microfilarial density and worm burden in *Onchocerca volvulus* infections in humans has been investigated, yet not fully understood. It has been observed that the microfilariae burden increases with host age, suggesting a development of immunological tolerance, while the density of adult parasites correlating with the microfilariae burden has not been confirmed. Since a purely age-related decline in host responsiveness is not shown, it is likely that immunological responsiveness against Mf diminishes as a consequence of cumulative parasite experience, pointing to immunological habituation or tolerance to the parasite (36).

All four cases presented here feature high microfilaremia in animals with severe co-morbidity and suspected immunodeficiency-related conditions. Helminths are known to suppress the host immune response in order to establish infection. For instance, it has been recently reported that D. repens may influence the host immune response by inducing a state of chronic in the canine host (29). It is possible that patients with high Mf counts may represent a population with pre-existing immunosuppression and are unable to naturally control or respond to the parasites. An improved screening and monitoring to determine the impact of chronic immunodeficiency disease on subcutaneous dirofilariosis and its correlation with neoplastic processes should be taken into consideration by investigators and practitioners for better understanding of *D. repens* pathogenicity.

## Data Availability Statement

All datasets generated for this study are included in the article/supplementary material.

## Ethics Statement

Ethical approval was not required for the animal study, because no interventions outside routine care and diagnostics were performed (as per Resolution Number 22/2006 of the National Commission for the Ethics of Experiments on Animals, 7th November 2006 and Act of 15th January 2015 on the protection of animals used for scientific purposes). Written informed consent was obtained from the owners for the participation of their animals in this study.

## Author Contributions

MW analyzed the data and wrote the manuscript. MS-F performed the histopathological examination of internal organs of the first case. MK, ED, MW, and MS-F critically revised the manuscript. All authors read and approved the final version of the manuscript.

## Conflict of Interest

The authors declare that the research was conducted in the absence of any commercial or financial relationships that could be construed as a potential conflict of interest.

## Footnotes

^1^https://www.esda.vet/wp-content/uploads/2017/11/GUIDELINES-FOR-CLINICAL-MANAGEMENT-OF-SUBCUTANEOUS-DIROFILARIOSIS-IN-DOGS-AND-CATS.pdf.
